# *Global Health: Science and Practice* … 5 Years In

**DOI:** 10.9745/GHSP-D-18-00196

**Published:** 2018-06-27

**Authors:** Ruwaida M. Salem, Steve Hodgins

**Affiliations:** a Associate Managing Editor, Global Health: Science and Practice Journal, Baltimore, MD, USA.; b Editor-in-Chief, Global Health: Science and Practice Journal, and Associate Professor, School of Public Health, University of Alberta, Edmonton, Alberta, Canada.

## Abstract

Five years after launching *Global Health: Science and Practice*, we are seeing signs that we are helping to fill an important gap in program-related evidence. Looking forward, we seek to offer better coverage for topics that are relatively neglected in the global health literature and to publish more papers by authors based in low- and middle-income countries. We invite authors to submit manuscripts on global health programs grounded in evidence from research, evaluation, monitoring data, or experiential knowledge, and encourage readers to access and share our free articles to find scalable approaches and important lessons to inform programs and policy.

See also related infographic.

In the face of wide disparities in population health outcomes between high-income and low- and middle-income countries, there is a strong imperative to achieve and sustain large gains in population health. “Science,” in the sense of context-free evidence for the efficacy of clinical interventions that is typically reported in the peer-reviewed health literature, is certainly an important input to strategies to achieve such population impact. But even if we've identified an intervention for which there is sound evidence of efficacy, how can we ensure the effectiveness of delivery of such an intervention at scale, under real-world conditions? Such questions are addressed to some degree in the gray literature, but more often than not project reports lack rigor. This can result in global health program managers, policy makers, and donors reinventing the wheel, and failing to draw lessons from relevant experience elsewhere.

## FILLING THE PROGRAM EVIDENCE GAP

We launched *Global Health: Science and Practice* (GHSP) 5 years ago because the United States Agency for International Development and the Johns Hopkins Center for Communication Programs recognized an important gap in program-relevant evidence. Our vision was to enable the global health community to better share important lessons arising from their program experience, and to do so with sufficient rigor. We also wanted to contribute to the development of new norms on how to study and document program implementation issues, with a view to informing global health policy and practice.

One of the ways that GHSP differs from most other journals is that we encourage authors not only to share the overall results or impact of their programs on health outcomes, but also to provide detail on *how* they implemented their programs and important contextual factors that may have interacted with features of their program efforts (Box). With such information, readers in other settings can make more fully informed judgements on the possible relevance of findings to their own settings and populations.

BOX **Attributes of GHSP**

- Focus on programs implemented under real-world conditions, with specifics on the “how” of implementation
- Details on the context in which the program was implemented that might influence implementation elsewhere
- Emphasis on scalable approaches with high-impact potential
- Insightful analysis grounded in evidence—whether from research studies, program evaluations, monitoring data, or observational and experiential knowledge

**FIGURE 1:**
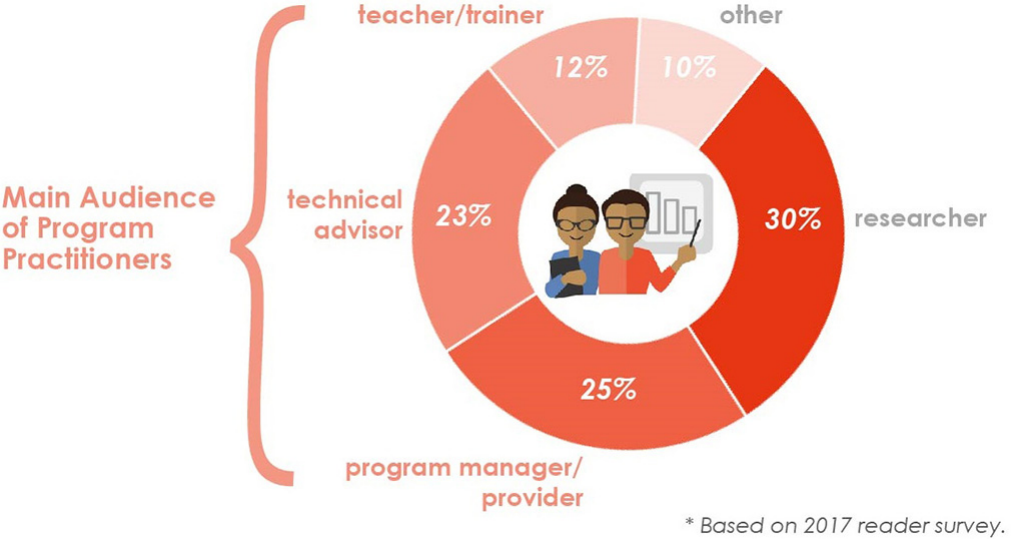
Job Function of GHSP Readers Source: “Celebrating 5 Years of Success” infographic: http://www.ghspjournal.org/5year-anniversary.

**FIGURE 2:**
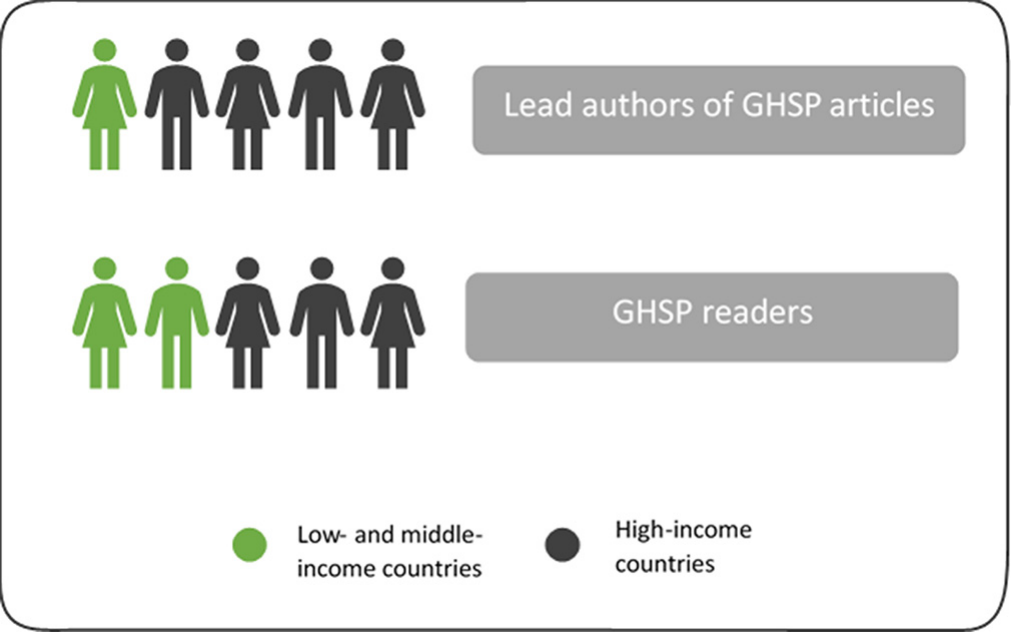
Current Reach of GHSP Into Low- and Middle-Income Countries Source: Data on lead authors from administrative data and on GHSP readers from Google Analytics data (total sessions from March 2013 through April 2017).

Another characteristic that differentiates us from most other journals serving the global health community is that we prioritize offering program implementers themselves a platform to share their valuable program experience. Such on-the-ground program practitioners often have less publishing experience than most of those submitting to scholarly journals, so we provide mentorship at different stages to coach them through the publication process. With no article-processing fees, we offer this support free of charge for authors, and we also provide readers free full-text access to all our articles—thus eliminating financial barriers to sharing and accessing important lessons from programs. In a GHSP reader survey conducted in 2017, 60% of respondents identified themselves as program managers, providers, technical advisors, teachers, or trainers, suggesting we are reaching our main audience of program practitioners (Figure 1). Furthermore, approximately 1 in 5 lead authors of GHSP published articles and 2 in 5 of our readers come from low- and middle-income countries (Figure 2).

## What Is the Journal Achieving?

Since launching 5 years ago, GHSP has published 45 editorials and 273 original articles, commentaries, viewpoints, field action reports, and other types of articles, as well as 2 supplements. In total, these articles have been accessed 1.2 million times, with our most popular articles covering a range of global health topics including digital health,^1^^,^^2^ adolescent sexual and reproductive health,^3^ tuberculosis,^4^ immunization,^5^ family planning,^6^ health promotion,^7^ and health systems.^8^ The number of readers accessing our articles has increased each year, from about 60,000 full-text accesses when we launched in 2013 to nearly 375,000 in 2017. And many of our articles are being cited in the peer-reviewed literature. Scopus calculates that each GHSP article receives, on average, 1.24 citations (using data from May 31, 2017).^8^ In comparison, each article in *Global Public Health* receives, on average, 1.60 citations, and in the *Bulletin of the World Health Organization* 3.09 citations—these journals were launched in 2006 and 1948, respectively.^9^

Clearly, for any journal, citations are an important measure of impact. However, citations in the peer-reviewed literature are also something of an echo chamber; the literature references itself, whether or not it is having useful impact in the real world. A central part of our ambition at GHSP is to serve the policy and program community, providing them with rigorously grounded but practical lessons arising from global public health program work, i.e., “practice-based evidence.”^10^^,^^11^ More important to us than number of citations in the peer-reviewed literature is seeing lessons documented through GHSP picked up and influencing policy and practice.

### Impact on Practice

**FIGURE 3:**
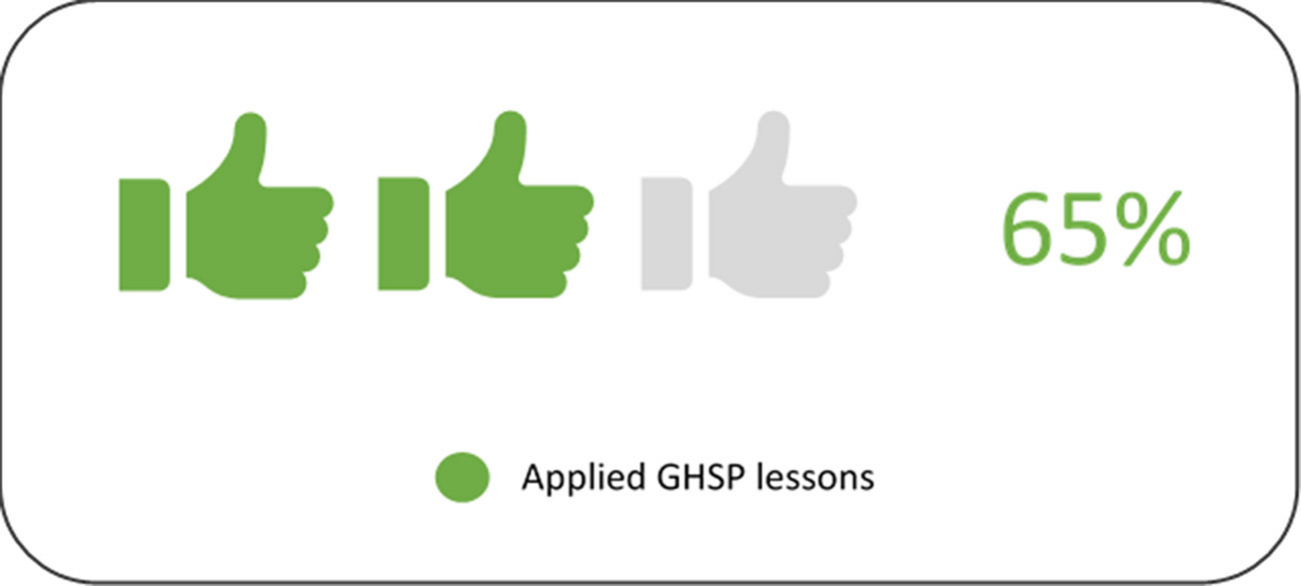
Use of GHSP Lessons by Readers Source: 2017 GHSP reader survey.

We have some indication that insight captured in GHSP authors' work is being picked up and applied in other contexts. In a recent survey of our readers, about two-thirds reported they've applied lessons from GHSP articles in their own public health practice (Figure 3). Of those, 42% said they used lessons from GHSP to design new programs or improve existing ones. For example, a technical advisor in Nigeria made use of an article from GHSP on task-shifting provision of contraceptive implants to community health extension workers^12^ to advocate to the national reproductive health technical working group to allow lower-level health workers to perform family planning procedures. In Rwanda, a teacher used lessons drawn from an article on trends in HIV prevalence and sexual behaviors in Burkina Faso^13^ to develop an approach to sex and drug abuse-related behavior change communication work with youth. A quarter of our surveyed readers who report having used GHSP articles to inform their programs said they used the articles to create or revise training or educational materials. For instance, a clinician in India used a GHSP article on operational challenges to Ebola case investigation in Sierra Leone^14^ to develop a mock exercise for university students to explore how to investigate an Ebola case.

## LOOKING AHEAD

In the coming years, we are looking to attract more readers and authors across a broad range of global health topics, including non-communicable diseases and injury, malaria, nutrition, water and sanitation, mental health, and HIV/AIDS, who share our common goal of improving global health programs and population health outcomes. We especially want to continue expanding our reach into low- and middle-income countries, to help people on the ground who are actually designing and implementing programs share what they've been learning with each other and develop more effective programs. To facilitate that, we seek to build partnerships with institutions in low- and middle-income countries to learn from their experiences while helping to strengthen their capacity in scholarly publishing. We will also continue to expand our reach by, for example, linking with other major indexing services beyond MEDLINE, PubMed, and Scopus, in which we're already included, and to explore adding new high-value features and services, such as webinars, online journal clubs, and translation of abstracts.

We seek to build partnerships with institutions in low- and middle-income countries to learn from their experiences while helping to strengthen their capacity in scholarly publishing.

Most importantly, we will keep our eye on what we set out to do 5 years ago—“to find principles and lessons learned that are systematic, replicable, and applicable in other settings”^15^ to contribute to stronger, more effective health programs delivered at scale and to accelerate improvements in population health status.
